# Zoledronic acid ameliorates the effects of secondary osteoporosis in rheumatoid arthritis patients

**DOI:** 10.1186/s13018-019-1492-3

**Published:** 2019-12-10

**Authors:** Jun Xie, Shaohua Li, Lianbo Xiao, Guilin Ouyang, Lin Zheng, Yubiao Gu, Chengxin Gao, Xiuwei Han

**Affiliations:** 1 Department of Orthopedics, The Tenth People’s Hospital Affiliated to Nanjing Medical University, No. 301 Yanchangzhong Road, Shanghai, 200000 China; 2 Department of Joint Orthopedics, Guanghua Hospital Affiliated to Shanghai University of Traditional Chinese Medicine, No. 504 Xinhua Road, Shanghai, 200052 China

**Keywords:** Zoledronic acid, Inflammation, Osteoporosis, Rheumatoid arthritis

## Abstract

**Background:**

Secondary osteoporosis may occur in patients with rheumatoid arthritis (RA), causing irreversible joint damage and disability. Bisphosphonates, the recently developed bone resorption inhibitors, have demonstrated significant therapeutic effects on senile and postmenopausal osteoporosis. This study evaluated the efficacy and safety of zoledronic acid (ZOL), with or without methotrexate (MTX), for the prevention and treatment of bone destruction in RA patients.

**Methods:**

We recruited 66 RA patients with symptoms of secondary osteoporosis. They were randomized into three treatment groups—combined treatment with MTX and ZOL, ZOL monotherapy, or MTX monotherapy—in two consecutive 6-month periods. The participants were followed for 12 months. At the end of each treatment period, improvement in disease activity, bone destruction, and fracture risk were evaluated.

**Results:**

Combined treatment with ZOL and MTX had significantly better clinical efficacy compared with either ZOL or MTX monotherapy (*P* < 0.05). The combination significantly improved the lumbar spine and hip BMD and reduced FRAX scores, suggesting that ZOL combined with MTX reduces bone loss and risk of hip fracture in RA patients with secondary osteoporosis.

**Conclusion:**

ZOL has a synergistic effect when combined with MTX, inhibiting RA disease activity, reducing fracture risk, and improving quality of life in RA patients with secondary osteoporosis.

**Trial registration:**

Chinese Clinical Trial Registry, ChiCTR1800019290. Registered 3 November 2018–Retrospective registered, http://www.chictr.org.cn/showproj.aspx?proj = 31758

## Introduction

Rheumatoid arthritis (RA) is a chronic systemic autoimmune disease with an estimated global prevalence of 1 to 3% [1–3]. In China, nearly 5 million people have RA [4, 5], causing a significant economic and social burden. RA is characterized by chronic symmetrical and progressive polyarthritis accompanied by pain, stiffness, and swelling in the patients’ joints. Pathological changes in the synovial membrane of the joint include chronic inflammation, hyperplasia, and vasospasm, as well as the invasion of the articular cartilage, subchondral bone, ligaments, and tendons. These changes cause the destruction of the articular cartilage, bone, and the joint capsule, eventually leading to irreversible joint deformity and loss of function and a disability rate as high as 50%.

The chronic local and systemic inflammation observed in RA patients is often accompanied by multiorgan dysfunction. Roughly 15 to 36% of RA patients develop osteoporosis [6, 7] as a complication as early as 2 years after disease onset, increasing risks of local and systemic bone erosion, decreased bone density, and increased fracture risk [8]. Methotrexate (MTX), a potent disease-modifying antirheumatic drug (DMARD) and a preferred treatment for RA [2], has little effect on RA-associated systemic osteoporosis [9].

Bisphosphonates, a family of drugs that have been widely used as bone resorption inhibitors for osteoporosis for decades [10], inhibit calcium hydroxyphosphate dissolution and osteolysis [11]. Recently, a third generation of bisphosphonates, which includes commonly used alendronate, risedronate, and zoledronic acid (ZOL), has been developed. These drugs have been shown to inhibit bone resorption of osteoclasts, reduce bone turnover rate, increase bone strength, protect articular cartilage, and have anti-inflammatory effects [10]. These therapeutic effects have been demonstrated in senile and postmenopausal osteoporosis, but results from studies of RA-associated osteoporosis are inconclusive [12, 13]. In this study, we aimed to determine the efficacy of ZOL alone or in combination with MTX for the prevention and treatment of bone destruction in RA patients.

## Materials and methods

### Study population

The participants were recruited from patients diagnosed with RA-associated secondary osteoporosis at the outpatient clinic of Shanghai Guanghua Hospital of Integrated Traditional Chinese and Western Medicine from June 2013 to December 2015. Eligible participants were defined as those who had been clearly diagnosed with RA and had either a *T* value ≤ − 2.5 of L1-L4 and hip bone mineral density (BMD) measurements plus obvious low back pain, night convulsions, and limited life function symptoms; or a *T* value between − 1.5 and − 2.5 of L1-L4 and hip BMD measurements plus obvious low back pain, night convulsions, limited life function symptoms, and a history of one or more fractures. Exclusion criteria included (a) severe primary diseases in the cardiovascular, cerebrovascular, liver, kidney, or hematopoietic systems, consumptive chronic diseases, or mental illness; (b) hypercalcemia, fresh fractures, liver or kidney dysfunction, thyroid-related diseases, such as hypothyroidism and hyperthyroidism, or malignant tumors; (c) other joint-involving rheumatic diseases, such as systemic lupus erythematosus, dermatomyositis, Sjogren’s syndrome, ankylosing spondylitis, and gout; (d) allergy to ZOL or a history of other allergies; (e) pregnancy, breastfeeding, or planning for pregnancy in the near future; (f) currently participating in other clinical trials; and (g) lack of consent to receive treatment as directed in this protocol.

### Experimental design

**Fig. 1:**
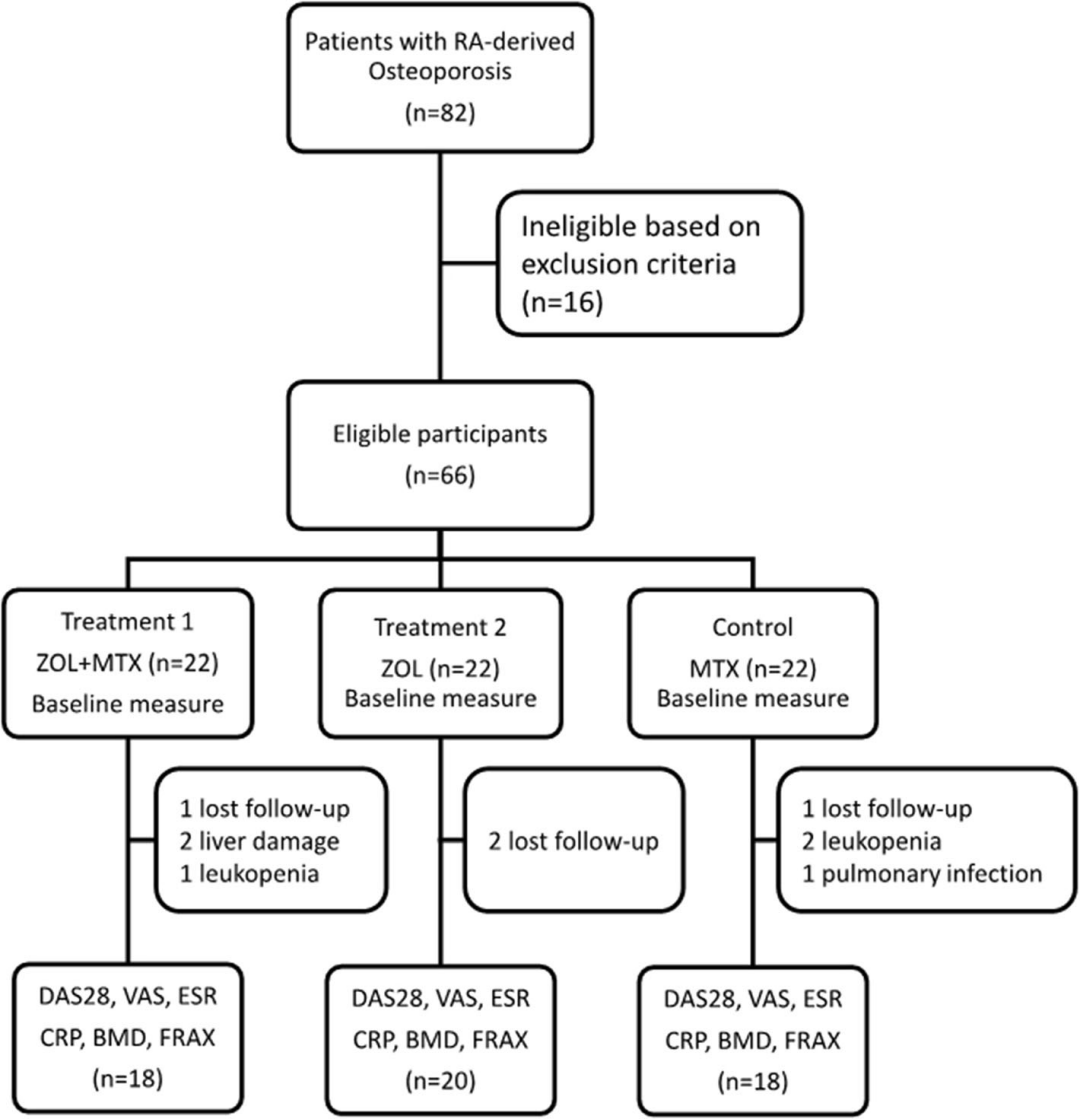
Sixty-six eligible participants were randomized into three treatment groups and followed for 1 year for clinical outcomes on DAS28, VAS, ESR, CRP, BMD, and FRAX.

Participants were randomized into three groups: combined treatment with ZOL and MTX, ZOL monotherapy, MTX monotherapy (control) (Fig. 1). A random number table (Additional file 1: Table S1) was generated along with random cards using the SAS8.0 software package. For each random card, a serial number, a random number, and a group number were generated and sealed in an envelope marked with the serial number. Eligible participants were enrolled in the order of clinical entry. Physicians obtained and opened an envelope with a serial number and treated patients as specified on the card.

Participants in the ZOL treatment groups received infusions of 0.5 mL of 0.9% NaCl followed by 1.5 mg of sodium zoledronate (Swiss Novartis Pharmaceutical Co., Ltd.) intravenously at a controlled rate with instillation time over 30 min and then 100 mL of 0.9% NaCl afterwards. Participants in the MTX treatment groups received 7.5 to 15 mg methotrexate tablets (2.5 mg/tablet; Shanghai Xinyi Pharmaceutical Co., Ltd.) orally once a week after meals. Additionally, each participant received a 600-mg calcium supplement (Wyeth Pharmaceutical Co., Ltd., Suzhou, China) and soft capsules containing 0.25 to 0.5 μg alfacalcidol (Teva Pharmaceutical Industries, Ltd., Israel) once daily. Participants completed two consecutive courses of treatment, each lasting 6 months, for a total follow-up of 12 months.

For patients taking NSAIDs at enrollment, the NSAID dose was stable for at least 4 weeks before treatment. For those who had been receiving MTX treatment at enrollment, they were left for at least 3 months of treatment and a stable dose for at least 4 weeks before entering the study. A 2-month washout period before treatment was used for those taking oral glucocorticoids at the time of enrollment. A 3-month washout period was used for those who had received other remission medications, including chloroquine, penicillamine, or sulfasalazine.

### Diagnostic criteria

According to the Classification Criteria for Rheumatoid Arthritis revised in 2003 by the American Rheumatology Association [14], the diagnostic criteria for RA includes the following: morning stiffness for at least 1 h for over 6 weeks; three or more joint areas of arthritis for over 6 weeks; wrists, metacarpophalangeal joint, or proximal interphalangeal arthritis for over 6 weeks; symmetric arthritis for over 6 weeks; subcutaneous nodules; radiographic changes on hands; and rheumatoid factor positive with titers higher than 1:32. Patients with four or more of the above seven items were diagnosed as having RA.

Osteoporosis was diagnosed using the *T* value from a BMD test according to the WHO standard deviation diagnostic method [15]. Patients were classified as “normal” with a *T* value not less than − 1.0 SD, “reduced bone mass” with a value between − 1.0 and − 2.5 SD, “osteoporosis” with a value less than − 2.5 SD, or “severe osteoporosis” with a value less than − 2.5 SD and one or more fractures.

### Outcome assessment

All participants were assessed before and after treatment for morning stiffness, patient self-evaluation and physician evaluation, C-reactive protein (CRP) level, erythrocyte sedimentation rate (ESR), BMD scores, and calculated FRAX scores. Disease activity score-28 (DAS28) was calculated with the 28-joint tender joint and swollen joint count, ESR, and health assessment [14]. A visual analog scale (VAS) was used to determine pain [16]. BMD scores of lumbar vertebrae 1 to 4 and the femoral neck, ward area, and large trochanter (GT), which were measured using a GE Lunar Prodigy dual-energy X-ray absorptiometry scanner, were expressed in g/cm^2^. FRAX scores were calculated using the FRAX interface (http://www.shef.ac.uk/FRAX/), incorporating collected data on fracture risk factors, to predict the probability of hip fracture in the next 10 years.

### Safety assessment

Serum hemoglobin, white blood cell count, platelet count, alanine aminotransferase, urea nitrogen, creatinine, routine urine routine, and routine stool were collected before and after each study period. Patients with abnormal results for any of these variables at the end of the study were followed until they returned to normal or clinically stable.

### Statistical analysis

The data were analyzed on SPSS 19.0 statistical software. Count data were described as frequency or composition ratio, and their changes before and after treatment were analyzed by a chi-square (*χ*^2^) or non-parametric test. Continuous variables were expressed as mean ± SD. An independent *T* test was used for comparison between two groups with assumed normal distribution and equal variances; a repeated measures data analysis was used for comparison between groups with measurements at multiple time points; and an LSD-*T* test was performed for further comparison between groups. A rank sum test was used on data that did not fit to the normal distribution or when the variances were not equal. All statistical tests were considered two-sided; *P* values < 0.05 were considered statistically significant.

## Results

### Baseline characteristics of study participants

Sixty-six participants were enrolled in the study and randomized into combined ZOL and MTX treatment, ZOL monotherapy, or MTX monotherapy groups (Fig. 1). During treatment, there were four cases of dropout from the combined treatment group (one due to loss to follow-up, one due to leukopenia, and two due to liver damage), with 18 patients completing the trial; there were two cases of dropout from the ZOL monotherapy group (both due to loss to follow-up), with 20 patients completing the trial; and there were four cases of dropout from the MTX monotherapy group (one due to pulmonary infection, one due to loss to follow-up, and two due to leukopenia), with 18 patients completing the trial. In total 56 of them completed the study and were included in the analysis. In the combined group, there were 4 females and 14 males with 95%CI of age at 58.6 ± 4.8 and DAS28 at 5.98 ± 1.25; in ZOL group, there were 4 females and 16 males with age at 57.2 ± 5.1 and DAS28 at 5.72 ± 1.35; in MTX group, there were 3 females and 15 males with age at 56.3 ± 4.5 and DAS28 at 6.06 ± 1.28. There were no significant differences in the distribution of age, sex, and DAS28 between any two groups (*P* > 0.05 for all pair-wise comparisons).

### Changes in main clinical outcomes with treatment

Participants received two consecutive courses of treatment and were evaluated for the main clinical outcomes before and after treatment. An improvement in clinical symptoms, demonstrated with decreased scores in morning stiffness and VAS and DAS28 scores, was observed in all groups after the treatment (data not shown). Improvement in ESR and CRP was observed with combined treatment and MTX monotherapy (Table 1). Between groups, combined treatment resulted in greater improvement in morning stiffness compared with ZOL monotherapy after 6 months of treatment (*P* < 0.05). The combination provided greater improvement in VAS score compared with MTX monotherapy after 6 months of treatment and compared with both ZOL and MTX monotherapy after 12 months (*P* < 0.05) (Table 1). Improvement in ESR was greater with combination treatment than with ZOL or MTX monotherapy after both 6 and 12 months (Table 1). Effects on morning stiffness, CRP, and other clinical indicators did not differ between the ZOL and MTX monotherapy groups (*P* > 0.05 for all). Overall, combined treatment was more effective than ZOL or MTX alone.

**Table 1:** Changes of the main clinical outcomes before and after treatment (mean ± sd)

	Time	Combined	p*	ZOL	p*	MTX	p*
Morning stiffness (min)	0 M	92.21 ± 47.69	–	81.29 ± 35.56	–	89.97 ± 36.42	–
	6 M	21.34 ± 16.10^△^	< 0.001	71.27 ± 14.89^△^	0.371	44.15 ± 13.99	0.021
	12 M	13.09 ± 14.43	< 0.001	42.69 ± 13.56^△^	0.019	25.19 ± 14.21	< 0.001
A.1.1.1.1.1.1. VAS (mm)	0 M	64.36 ± 18.29	–	63.94 ± 17.83	–	65.28 ± 17.27	–
	6 M	37.90 ± 14.04^△^	< 0.001	43.02 ± 16.20	0.040	40.08 ± 15.88	0.048
	12 M	29.23 ± 16.13^△^	< 0.001	37.02 ± 17.89^△^	< 0.001	38.72 ± 17.25^△^	< 0.001
A.1.1.1.1.1.2. ESR(mm/h)	0 M	49.87 ± 27.68	–	50.43 ± 34.78	–	52.25 ± 37.96	–
	6 M	30.61 ± 21.56	0.022	40.25 ± 28.75^△^	0.467	37.21 ± 25.47^△^	0.038
	12 M	23.58 ± 17.87	0.008	38.67 ± 22.87^△^	0.363	36.24 ± 21.46^△^	0.023
A.1.1.1.1.1.3. CRP(mg/L)	0 M	25.14 ± 22.23	–	23.67 ± 27.35	–	26.21 ± 28.38	–
	6 M	13.57 ± 15.89	0.031	18.52 ± 29.51	0.077	15.39 ± 26.29	0.035
	12 M	8.60 ± 12.27^△^	0.029	18.78 ± 23.38^△^	0.079	11.02 ± 20.12	0.019
A.1.1.1.1.1.4. DAS28	0 M	7.21 ± 1.27	–	7.17 ± 1.21	–	6.36 ± 1.45	–
	6 M	4.89 ± 1.26^**^	< 0.001	5.11 ± 1.33	0.036	4.27 ± 1.31	0.032
	12 M	4.17 ± 1.21^**^	< 0.001	4.67 ± 1.28	0.019	4.12 ± 1.27	0.021

Note: *VAS* visual analog scale, *ESR* erythrocyte sedimentation rate, *CRP* C-reactive protein, *DAS 28* disease activity scores-28; *p values indicate the comparison with that before treatment within the same group; △ indicates *p* < 0.05 for the comparison between two groups at the same time point on their difference over the treatment

### Prognosis of osteoporosis after treatment

Participants were further evaluated for osteoporosis at the end of each treatment regimen. In the BMD assessment summarized in Table 2, a significant improvement in bone mass at the lumbar spine and femoral neck after 6 months of treatment, and in all areas after 12 months of treatment (*P* < 0.05), was observed with combination therapy, with gains that were significantly greater compared with those seen with ZOL or MTX monotherapy (*P* < 0.05). Femoral bone volume significantly improved after 12 months of treatment with ZOL monotherapy (*P* < 0.05), but there was no difference between ZOL and MTX monotherapy (*P* > 0.05). In the fracture risk assessment, the FRAX score was significantly reduced with combined treatment after 6 months (*P* < 0.05), with a further decline observed after 12 months (*P* < 0.05); at 12 months, the FRAX score was significantly lower in the combination therapy group than in the ZOL and MTX monotherapy groups (*P* < 0.05) (Table 3). While the FRAX score was significantly lower in the ZOL monotherapy group after 12 months of treatment, there was no significant difference between the ZOL and MTX monotherapy groups at that time point (Table 3).

**Table 2:** BMD changes (g/cm^2^) in different parts before and after treatment

Area	Time	Treatment 1	p*	Treatment 2	p*	Control	p*
Lumbar1	A.1.1.1.1.1.5. 0 M	0.45 ± 0.25	–	0.49 ± 0.23	–	0.50 ± 0.18	–
	A.1.1.1.1.1.6. 6 M	0.50 ± 0.16	0.785	0.48 ± 0.22	0.983	0.52 ± 0.27	0.962
	A.1.1.1.1.1.7. 12 M	0.54 ± 0.19*	0.045	0.55 ± 0.23	0.680	0.55 ± 0.28	0.785
Lumbar2	A.1.1.1.1.1.8. 0 M	0.65 ± 0.17	–	0.61 ± 0.12	–	0.64 ± 0.16	–
	A.1.1.1.1.1.9. 6 M	0.78 ± 0.22*	0.041	0.66 ± 0.13	0.582	0.64 ± 0.17	0.992
	A.1.1.1.1.1.10. 12 M	0.72 ± 0.17*	0.049	0.67 ± 0.14	0.460	0.63 ± 0.14	0.980
	A.1.1.1.1.1.11. 0 M	0.69 ± 0.22	–	0.62 ± 0.24	–	0.70 ± 0.21	–
Lumbar3	A.1.1.1.1.1.12. 6 M	0.72 ± 0.13	0.914	0.69 ± 0.29	0.582	0.73 ± 0.16	0.914
	A.1.1.1.1.1.13. 12 M	0.81 ± 0.17*^△^	0.024	0.77 ± 0.25*	0.019	0.64 ± 0.27^△^	0.699
	A.1.1.1.1.1.14. 0 M	0.69 ± 0.18	–	0.65 ± 0.18	–	0.66 ± 0.16	–
Lumbar4	A.1.1.1.1.1.15. 6 M	0.74 ± 0.22	0.664	0.67 ± 0.15	0.929	0.65 ± 0.17	0.983
	A.1.1.1.1.1.16. 12 M	0.90 ± 0.21*^△^	0.011	0.68 ± 0.14△	0.848	0.63 ± 0.14△	0.862
	A.1.1.1.1.1.17. 0 M	0.54 ± 0.17	–	0.57 ± 0.14	–	0.57 ± 0.19	–
Ward area	A.1.1.1.1.1.18. 6 M	0.66 ± 0.14	0.076	0.54 ± 0.19	0.832	0.54 ± 0.14	0.848
	A.1.1.1.1.1.19. 12 M	0.80 ± 0.17*^△^	0.012	0.67 ± 0.17*△	0.036	0.50 ± 0.16△	0.411
Femoral neck	A.1.1.1.1.1.20. 0 M	0.68 ± 0.13	–	0.63 ± 0.14	–	0.62 ± 0.20	–
	A.1.1.1.1.1.21. 6 M	0.84 ± 0.15*	0.014	0.65 ± 0.17	0.926	0.65 ± 0.17	0.855
	A.1.1.1.1.1.22. 12 M	0.80 ± 0.21*^△^	0.047	0.69 ± 0.17*	0.502	0.60 ± 0.17△	0.933

Note: *p values indicate the comparison with that before treatment within the same group; △ indicates *p* < 0.05 for the comparison between two groups at the same time point on their difference over the treatment

**Table 3:** Changes of the risk of hip fracture

FRAX	Treatment 1 (*n* = 18)	p*	Treatment 2 (*n* = 20)	p*	Control (*n* = 18)	p*
Before treatment	5.76 ± 1.45	–	5.86 ± 1.59	–	5.83 ± 1.74	–
6 months	5.08 ± 2.17	0.047	5.98 ± 2.73	0.097	5.65 ± 1.67	0.145
12 months	4.34 ± 1.78^△^	0.035	5.44 ± 1.89^△^	0.072	5.64 ± 2.09	0.139

*p values indicate the comparison with that before treatment within the same group; △ indicates *p* < 0.05 for the comparison between two groups at the same time point on their difference over the treatment

### Safety evaluation

All participants were monitored in the safety analysis. Both ZOL and MTX monotherapy were well tolerated, with most side effects occurring within 48 h after drug administration; they included mild adverse reactions, mainly fever (3 last less than 24 h, 1 lasts between 24 and 48 h and 1 lasts longer than 48 h), joint pain (3 last less than 24 h and 3 last between 24 and 48 h), myalgia (4 last less than 24 h, 2 last between 24 and 48 h, and 1 lasts longer than 48 h), or gastrointestinal discomfort (1 lasts less than 24 h). No cases of inflammatory eye disease, jaw osteonecrosis, atrial fibrillation, or serum creatinine or creatinine clearance anomalies were observed. The abovementioned adverse reactions, often been found in patients treated with MTX [17], are most likely associated with the use of MTX, but not to zoledronate.

In summary, administration of zoledronic acid, combined with MTX, reduces RA disease activity, risk of fracture, and bone pain in patients with RA-derived secondary osteoporosis.

## Discussion

Bisphosphonates, which have a high affinity for bone mineral structures and an ability to chelate calcium ions, inhibit the formation, growth, and dissolution of hydroxyphosphonite crystals and their crystalline materials. The molecular mechanism underlying their action has recently been described [10]. While the simple and nitrogen-containing bisphosphonates may act through different pathways, both could cause osteoclast apoptosis and hence inhibit bone resorption. Thus, the bisphosphonate drugs such as disodium etidronate, pamidronate, alendronate sodium, risedronate sodium, and zoledronic acid are commonly used in osteoporosis treatment and prevention to increase bone density and reduce the risk of vertebral fractures. Interestingly, the nitrogen-containing bisphosphonates such as ZOL, by inhibiting the mevalonate pathway of osteoclasts, suppress downstream protein synthesis involving Rho, Ras, and Rab [10], a pathway associated with inflammation. Rho is an inflammation-related protein that promotes the migration of macrophages and lymphocytes to inflammatory tissues, which mediates an inflammatory response [18]. Therefore, ZOL treatment also helps lessening the RA symptoms.

RA and bone destruction have been considered two unrelated diseases of the immune system and the bone metabolism system, respectively. Thus, joint inflammation and bone destruction in RA patients have been treated as two major pathological manifestations and as separate therapeutic targets. In the clinic, RA patients typically receive hormones and/or DMARDs to control joint inflammation for symptom relief [2]. On the other hand, bisphosphonates have been utilized to reduce and prevent bone damage caused by RA or osteoporosis through inhibition of osteoclast activation and induction of osteoclast apoptosis [8, 10].

In recent years, data has accumulated to strongly suggest that the RANKL-RANK system may be involved in RA-mediated bone destruction. It has been known that maturation activation of osteoclasts is induced by RANKL, a critical step in developing both localized bone erosion and systemic osteoporosis [19]. Studies of the RA animal model have shown that the expression of RANKL is upregulated in inflammatory joints [20–24]; the level of secreted osteoprotegerin (OPG), a TNF receptor-related protein, is decreased; and the ratio of RANKL to OPG is positively correlated with osteoclast activity and local bone erosion. On the other hand, inhibition of RANKL significantly reduces bone erosion. For example, RANKL knockout mice are more resistant to arthritis-associated joint erosion and have fewer osteoclasts in joints [25], while in arthritic rats, OPG can inhibit bone erosion and osteoclast function and thus reduce the loss of bone mass around the joint [26].

These studies all point to the close relationship between RA inflammation and osteoporosis. Thus, a treatment regimen that targets osteoporosis alone may not be sufficient for the optimal therapeutic effect. In this study, we demonstrated that treatment with ZOL alone has little effect on RA-derived secondary osteoporosis, but that when it is combined with MTX it reduces both RA inflammation and bone destruction. Previous studies have shown that for women with postmenopausal RA, whose femoral bone density is correlated with the cross-sectional area of the proximal thigh muscle and the time since menopause, bisphosphonate treatment alone has no significant effect on the bone density of the femoral shaft [13]. On the contrary, in other studies from women with postmenopausal RA and a history of corticosteroid treatment, DAS28 score and bone density of the lumbar spine and femoral neck were significantly improved after 12 months of treatment with alendronate or risedronate along with the original RA treatment. Thus, for postmenopausal women with RA, combined treatment with bisphosphonates and anti-inflammatory medication can increase bone density and slow disease activity [27]. That is supported by another similar study, which showed that the use of bisphosphonates alone is not optimal for the prevention and treatment of secondary osteoporosis and femoral neck fracture, but when combined with statins, has a significant beneficial effect [28].

There is concern whether 12 months of follow-up time in our study is long enough for the evaluation of bone density changes. Several studies [29–31] reported that the bone density changes from 6 months to 2 years with relative treatments. Consistent with these findings, we observed significant improvement of bone mass with combined therapy and reduced FRAX score, starting from 6 month, followed by less significant effect with other two treatments (Table 3). Thus, 12 months of follow-up is sufficient to determine whether the treatment effect should exist. We also continue following up with patients to evaluate the long-term treatment effect, which will be discussed in the future.

In conclusion, inflammation is the most important factor associated with RA bone loss. The combination of methotrexate with bisphosphonate drugs under the premise of complete control of inflammation is the best treatment option for RA-associated secondary osteoporosis.

## Supplementary information

**Additional file 1:** Table S1. Random number table generated for zoledronate clinical trial

## Data Availability

The datasets used and/or analyzed during the current study are available from the corresponding author on reasonable request.

## Abbreviations

BMD: Bone mineral density
CRP: C-reactive protein
DAS28: Disease Activity Score-28
DMARD: Disease-modifying antirheumatic drug
MTX: Methotrexate
RA: Rheumatoid arthritis
VAS: Visual analog scale
ZOL: Zoledronic acid
