# Supplementary material for: Zoledronic acid ameliorates the effects of secondary osteoporosis in rheumatoid arthritis patients
Source: J Orthop Surg Res. 2019 Dec 10;14:421. doi: 10.1186/s13018-019-1492-3 (PMC6902494; doi:10.1186/s13018-019-1492-3)
Supplement: Supplementary file 1 — Additional file 1: Table S1. Random number table generated for zoledronate clinical trial [file 13018_2019_1492_MOESM1_ESM.docx]

**Supplemental data**

Table 1. Random number table generated for zoledronate clinical trial

| 1A | 2B | 3A | 4C | 5C | 6B | 7C | 8A | 9C | 10B |
| --- | --- | --- | --- | --- | --- | --- | --- | --- | --- |
| 11B | 12A | 13A | 14B | 15B | 16B | 17C | 18A | 19C | 20C |
| 21C | 22B | 23B | 24C | 25C | 26A | 27A | 28B | 29A | 30A |
| 31B | 32C | 33A | 34A | 45B | 36C | 37B | 38C | 39A | 40A |
| 41C | 42C | 43B | 44B | 45A | 46B | 47C | 48A | 49B | 50A |
| 51C | 52B | 53B | 54A | 55C | 56A | 57B | 58A | 59C | 60C |
| 61C | 62B | 63A | 64A | 65B | 66C |  |  |  |  |

Note: seed=20121029
